# Designing Efficient Sinkhole Attack Detection Mechanism in Edge-Based IoT Deployment

**DOI:** 10.3390/s20051300

**Published:** 2020-02-27

**Authors:** Sumit Pundir, Mohammad Wazid, Devesh Pratap Singh, Ashok Kumar Das, Joel J. P. C. Rodrigues, Youngho Park

**Affiliations:** 1 Department of Computer Science and Engineering, Graphic Era Deemed to be University, Dehradun 248 002, India; sumitpundir.it@geu.ac.in (S.P.); wazidkec2005@gmail.com (M.W.); devesh.geu@gmail.com (D.P.S.); 2 Center for Security, Theory and Algorithmic Research, International Institute of Information Technology, Hyderabad 500 032, India; iitkgp.akdas@gmail.com or; 3 Federal University of Piauí (UFPI), 64049-550 Teresina-Pi, Brazil; joeljr@ieee.org; 4 Instituto de Telecomunicações, 1049-001 Lisbon, Portugal; 5 School of Electronics Engineering, Kyungpook National University, Daegu 41566, Korea

**Keywords:** sinkhole attack, internet of things (IoT), intrusion detection, edge computing, NS2 simulation, security

## Abstract

The sinkhole attack in an edge-based Internet of Things (IoT) environment (EIoT) can devastate and ruin the whole functioning of the communication. The sinkhole attacker nodes (SHAs) have some properties (for example, they first attract the other normal nodes for the shortest path to the destination and when normal nodes initiate the process of sending their packets through that path (i.e., via SHA), the attacker nodes start disrupting the traffic flow of the network). In the presence of SHAs, the destination (for example, sink node i.e., gateway/base station) does not receive the required information or it may receive partial or modified information. This results in reduction of the network performance and degradation in efficiency and reliability of the communication. In the presence of such an attack, the throughput decreases, end-to-end delay increases and packet delivery ratio decreases. Moreover, it may harm other network performance parameters. Hence, it becomes extremely essential to provide an effective and competent scheme to mitigate this attack in EIoT. In this paper, an intrusion detection scheme to protect EIoT environment against sinkhole attack is proposed, which is named as SAD-EIoT. In SAD-EIoT, the resource rich edge nodes (edge servers) perform the detection of different types of sinkhole attacker nodes with the help of exchanging messages. The practical demonstration of SAD-EIoT is also provided using the well known NS2 simulator to compute the various performance parameters. Additionally, the security analysis of SAD-EIoT is conducted to prove its resiliency against various types of SHAs. SAD-EIoT achieves around 95.83% detection rate and 1.03% false positive rate, which are considerably better than other related existing schemes. Apart from those, SAD-EIoT is proficient with respect to computation and communication costs. Eventually, SAD-EIoT will be a suitable match for those applications which can be used in critical and sensitive operations (for example, surveillance, security and monitoring systems).

## 1. Introduction

**Figure 1:**
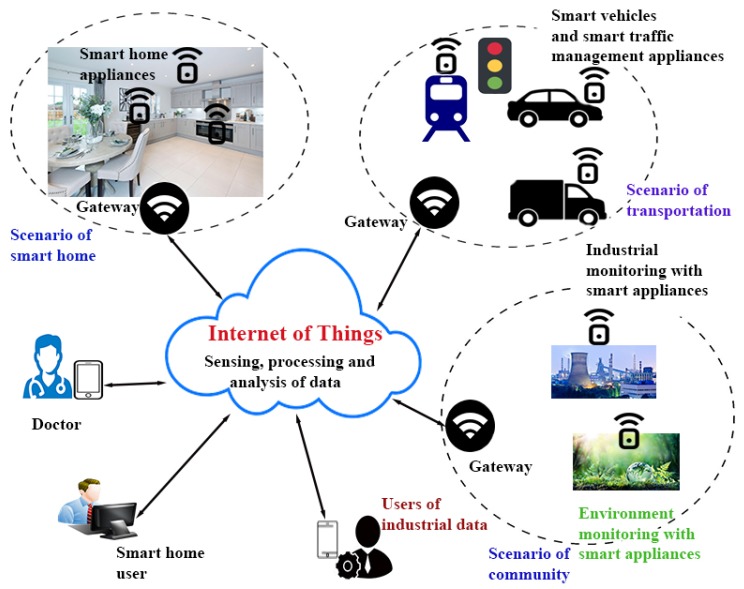
Generic Internet of Things (IoT) architecture (adapted from [1]).

Internet of Things (IoT) objects include smart sensors (devices) which are capable of gathering and transmitting the sensing information in an IoT network where the human involvement is minimally required. In a generalized IoT architecture presented in Figure 1 [1,2] consists of various scenarios like smart home, smart transportation and smart community. All these scenarios are installed with smart devices such as smart home appliances, smart traffic management appliances and smart environment monitoring devices. These devices are placed and installed in order to communicate with other heterogeneous devices over the Internet via the gateway node(s) (GWN) where the traffic goes out and comes in. In this scenario, various users (i.e., smart home users) use the GWNs to access the real-time information via smart devices deployed in the network [1,3,4]. IoT based applications become the essential requirement of the society as they provide people a dependable, responsive and ingenious network connectivity which helps in controlling the remote smart IoT devices in a real time fashion.

In an edge-based communication network, the data is processed near the edge (i.e., edge device) where the data is being generated unlike the centralized data-processing facility. This further improves response time of the network and also saves the bandwidth. In edge computing, there is a corner (i.e., edge) where the data traffic goes out and comes in, which is also named as edge router (device or node) or simply a gateway. In an edge-based IoT communication environment, edge node performs heavy computations on data on behalf of the smart IoT devices which have limited computational, communication and storage resources. The data analysis is performed near the sensing devices which speed up the process of data analysis and further reduce the decision making time [5,6]. Edge-based IoT communication environment (EIoT) is better than the cloud-based architecture because of its enhanced and improved performance with moderate cost. EIoT may have different applications such as smart cities, smart health care system and smart environment monitoring like the cloud computing communication environment. However, at the same time, EIoT suffers from several privacy and security problems. EIoT consists of different devices such as edge router (gateway node), various users and smart devices. Most of the time interested users can access real-time data possessed by the smart devices [3,5,6,7,8]. For such kind of communication environment, edge computing provides additional benefits such as “enhanced security, decreased bandwidth and reduced latency”. Henceforth, it is promising communication technology for different IoT applications.

**Figure 2:**
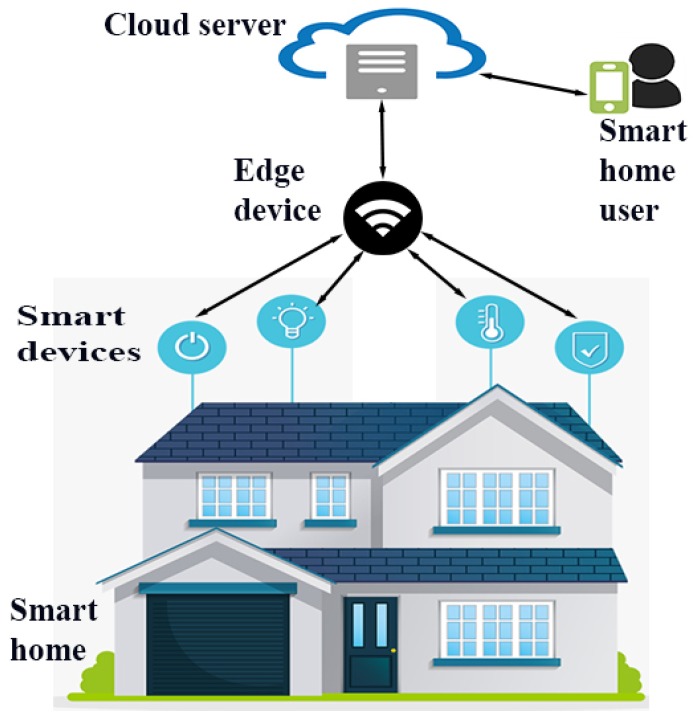
Edge-based IoT architecture for smart home scenario.

Edge-based IoT architecture for smart home scenario is presented in Figure 2. In this scenario, certain smart devices are deployed to monitor and support the day-to-day activities inside a home. The smart devices (i.e., IoT sensors) sense, process and send the data of home appliances to the nearby node (edge device). The edge server receives and further processes the data and forwards it to the cloud server. The authenticated (genuine) smart home users can access the data of the smart home through the cloud servers. The advantage of this architecture is that the resource rich edge devices can do processing on behalf of resource constrained smart devices. Furthermore, edge devices can also be utilized for other types of tasks, such as for the detection of intrusion in the network as they are powerful devices (resource rich nodes) of the network. Hence, the same approach is followed in this paper.

**Figure 3:**
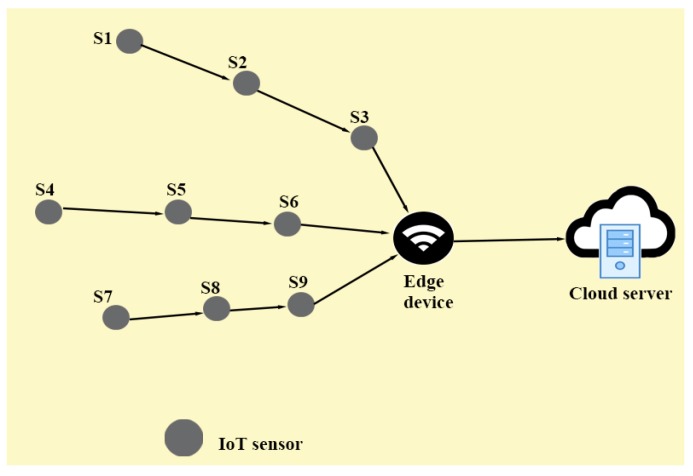
Edge-based Internet of Things (IoT) environment (EIoT)-communication under the normal flow of traffic.

Additionally, Figure 3 is also provided which consists of different kinds of devices (for example, edge router (gateway) and smart devices, i.e., IoT sensors and cloud servers). The task of an IoT sensor is to sense, process and transmit the data of environmental phenomena (i.e., humidity level, temperature, pressure, etc.) to the edge node. IoT smart devices (IoT sensors) are resource-constrained with limited memory, battery backup and processing capability, whereas the edge node is rich in resources (in terms of memory, battery power to sense and process the data and long communication ranges). Then an edge node processes and analyzes the received data and after that it transmits the processed data to the remote server(s) over the cloud. However, such type of communication environment is vulnerable to various security threats which lead to significant security problems as different attacks (for instance, routing attacks, sybil attack, data leakage, replaying of old messages, man-in-the middle, impersonation, physical capturing of smart devices, password guessing and privileged insider attacks) are possible [1,3,6,9].

Zhao et al. [10] designed a mechanism to detect location injection attacks (LIAs), called ILLIA. ILLIA is based on the “k-anonymity-based privacy preservation against LIA in continuous location-based services (LBSs) queries”. They emphasized that the attackers attempt to attack some particular users who they are interested in. Such type of user is referred to as high-risk user. ILLIA has the ability to protect LIA without having advance knowledge of how fake locations are exploited. At the same time, ILLIA also maintains high quality of services.

Wang et al. [11] proposed another intrusion detection approach, known as MIASec. It provides “input data indistinguishability” and defends against membership inference attacks in “Machine Learning as a Service (MLaaS)”. In a “membership inference attack”, an attacker is provided with given black-box access to a target classifier and inclines to infer if a specific record is covered in the training set or not.

### 1.1. Attack Schema of Sinkhole Attack in Edge-based IoT Environment

**Figure 4:**
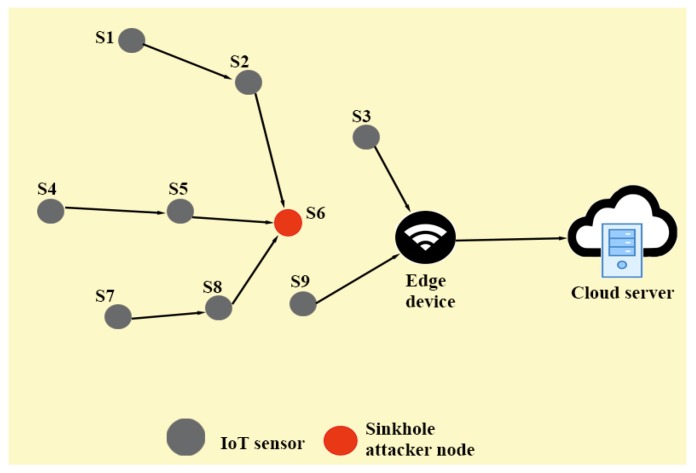
EIoT-communication in the case of sinkhole attack.

A possible scenario of sinkhole attack in EIoT environment provided in Figure 4 represents the flow of network traffic under the presence of SHAs. The communication scenario of EIoT under the normal flow of traffic has been presented in Figure 3. Each IoT smart device (i.e., IoT sensor) can sense and transmit the information to the destination (i.e., edge device/node) under the normal flow of traffic. After collecting the data from different IoT devices, an edge node can process and analyze the data and then transmit the information to the cloud server for further processing and storage.

An adversary, say A, can physically capture some IoT smart devices as the devices are installed (deployed) in an unattended (hostile) environment where 24×7 h physical monitoring may not be feasible. It further helps A to physically capture some nodes (IoT sensors). Thus, A can extract the stored sensitive information by applying power analysis attacks [12,13]. A can also install malicious sinkhole attack by launching script in the IoT devices which can execute the attack [14,15,16,17,18]. Then, A can deploy these malicious nodes in the target area to launch the required attack. When SHAs start working in the network, the confidential information may be leaked, delayed or lost [17], which can further trouble the entire functioning of the network. Therefore, a robust mechanism to defend against SHAs is required. Hence, intrusion detection technique for EIoT has become one of the primary research problems in recent years [14,15,16,17,19,20,21,22,23,24].

### 1.2. Goal of Designing Proposed Scheme

Sometimes IoT devices are installed in an “unattended (hostile) environment” (for example, smart security and surveillance), where the ceaseless physical monitoring of these devices [25,26,27,28] is not possible. A may take the advantage of lack of physical monitoring and captures some legitimate IoT sensor nodes. It is worth noticing that the SHAs have several interesting properties, such as they first attract the other normal nodes for the shortest path to the destination, and when normal nodes start sending their packets through that path (i.e., via SHA), the SHAs start disrupting the flow of the traffic. As a result, the SHAs may or may not forward the packets to the destination. Moreover, it may forward the packets selectively (for example, forwarding of packets of a particular network service (i.e., UDP packets) and restricting the others (i.e., TCP packets)). The packets are passed through the SHAs, which may delay, drop or modify the information inside these packets. An EIoT network consists of resource rich edge nodes (servers) and resource constrained sensing devices. The resource rich edge nodes can be used to detect the presence of attacker nodes. In our proposed architecture, the edge nodes are assumed to be the destination nodes, which receive the packets from the other neighbour nodes. In the presence of SHAs in EIoT, an edge node may not receive the information or it may receive modified or partially modified information. It then degrades the performance, efficiency and reliability of the communication happening in EIoT. In the presence of SHAs, there are various chances: (a) decrement in throughput of the network, (b) increment in end-to-end delay and (c) decrement in packet delivery ratio. The sinkhole attack has been investigated in recent years in wireless sensor networks and several solution were proposed. However, the chance of occurrence of sinkhole attacks in IoT environment is also high. The sinkhole attack detection techniques for Wireless Sensor Networks (WSNs) are not directly applicable in IoT. Therefore, a new scheme to mitigate sinkhole attacks in IoT is required. Hence, an effective intrusion detection scheme for protecting sinkhole attacks in an EIoT communication environment has been designed.

### 1.3. Attack Model

The widely followed “Dolev-Yao threat model (known as the DY model)” [29] can be used in the designing of SAD-EIoT, where “any two communicating entities communicate over an insecure public channel” [30]. Thus, the communication channel is treated as insecure and also the end-point entities (i.e., IoT sensors) are not considered to be trusted. Other possibilities of the sinkhole attack can also be considered. It is possible that an attacker A can physically capture some IoT smart devices (for example, IoT sensors) and take out the desired sensitive information from its memory [12,13]. A can clone new malicious nodes with sinkhole attack functionality by making use of extracted information. After the manufacturing of these malicious devices (i.e., SHAs), A can directly deploy them in the network [16,17,24]. As discussed in Section 1.1, under the successful execution of a sinkhole attack in EIoT, the data packets may get lost, dropped, delayed or modified. This may cause degradation in the performance of the communication in EIoT. Furthermore, this may cause the reduction in the throughput and packet delivery ratio of the network along with the increment in end-to-end delay. Hence, a strong intrusion detection mechanism to protect against the sinkhole attack is desirable in EIoT environment.

### 1.4. Research Contributions

The research contributions made in the proposed scheme are provided below.

- A new intrusion detection scheme for the detection of sinkhole attack in edge-based IoT environment (SAD-EIoT) has been proposed.
- SAD-EIoT is shown to be secure against sinkhole attacks in EIoT. It is validated through security analysis and also the results obtained through the NS2 simulation.
- Furthermore, SAD-EIoT is compared with other closely related existing techniques. The conducted comparison demonstrates that SAD-EIoT performs better than other existing techniques.

### 1.5. Structure of the Paper

The remainder of the paper is organised as follows. In Section 2, the background study of other related existing techniques is provided. Section 3 gives in-depth details of the designed intrusion detection scheme for sinkhole detection (SAD-EIoT) in EIoT environment. The research process and research methodology of SAD-EIoT is also explained here. Section 4 contains the details of various mathematical models. Section 5 contains the security analysis of SAD-EIoT, which proves that SAD-EIoT is competent and efficient enough to defend sinkhole attacks. The simulation study of SAD-EIoT using the widely-used NS2 tool is further provided in Section 6. Next, the performance comparison of SAD-EIoT with other closely related existing schemes is provided in Section 7. Finally, the work is concluded in Section 8.

## 2. Background

This section contains the background study of the various types of available intrusion detection techniques for Internet of Things and and wireless sensor network. The details are as follows.

Salehi et al. [31] designed a mechanism to detect sinkhole attack in Wireless Sensor Networks (WSNs). In the proposed mechanism, the attackers’ nodes are detected by considering the flow of packets in the network. First, the intrusion region is identified and then after collected data evaluation, the malicious nodes are identified. However, the presented mechanism raises the high false positive rate. Wang et al. [32] proposed a method to mitigate anomalies in a cluster-based WSN. A real time data monitoring system is used to analyse the data packets in the network, where any type of node (edge nodes, source nodes and sensors) can be compromised by an attacker node. Three types of detection schemes such as intelligent hybrid intrusion detection system (IDS), hybrid IDS and misuse IDS were used. The attacker nodes are identified by the misuse of data identification and anomaly detection. However, their implemented mechanism provides marginally improved detection rate.

Hamedheidari et al. [20] suggested a technique to defend WSN against the sinkhole attacks which uses mobile agent to inform the neighbours to prevent the attack. However, the overheads induced by the mobile agents reduce the performance of the network. Wang et al. [33] used “Gaussian distribution technique” for detection of intrusions. They discussed two methods to defend malicious nodes, called “single-sensing” and “multiple-sensing”.

Wang et al. [34] discussed the expected probability of “Intrusion Detection”. The provided solution uses parameters, such as node density, range in heterogeneous and homogeneous WSN for sensing anomalies. The calculated probability is used to analyse the efficiency and performance of the proposed method. To protect WSN from “misleading attackers in a multihop routing”, Zhan et al. [35] also suggested a framework named as“Trust-Aware Routing (TARF)”, which is capable of resolving the intrusions in dynamic sensor networks. Without making use of “time synchronisation and geographic location of the sensor nodes”, the trusted and energy saver routes can be identified.

Shin et al. [36] discussed various structures of “Wireless Industrial Sensor Networks (WISN)”. They proposed a hierarchical design to mitigate the attacks. “One-hop clustering” was the key point, which was utilised in the presented design. To mitigate the hierarchical anomalies, the logical protocols were used. Yu et al. [37] provided information about different kinds of intrusions and also the methods used to resolve them. A comprehensive literature survey was provided to highlight the existing standard and associated techniques in two different categories, which are for securing data and its routing.

Liu et al. [38] demonstrated an intrusion detection system to prevent sinkhole attack for the “Internet of Things (IoT)” communication. Their proposed scheme not only detects the SHAs but also bypasses the attack region by making use of routing mechanisms named as “hop-by-hop basis and multihop basis”. The location of the attacker nodes is also determined by their presented scheme. However, their scheme exhibits high computation and communication costs. Chen et al. [39] proposed a “Low-rate Denial of Service (LDoS)” attack mitigation technique which can be used for both WSN and IoT. They combined “Hilbert Huang transformation and trust evaluation” methods to find LDoS attacker nodes in WSN.

Fang et al. [40] examined the need for cyber security in “information-centric networking (ICN)”. They analysed the typical attack behaviours and defence methods. Furthermore, they presented a “Fast and Efficient Trust Management Scheme (FETMS)” for mitigating the On-Off attack with the help of communication trust, data trust and reputation values. Pongle et al. [41] investigated the possible attacks on “IPv6 over Low-Power Wireless Personal Area Networks (6LoWPAN)” and “IPv6 Routing Protocol for Low power and Lossy Networks (LLNs), called RPL”. They highlighted the possible techniques to mitigate these attacks. The emphasis was on protecting the RPL network from various attacks, such as Sybil, blackhole, wormhole and clone ID attacks.

Yang et al. [42] did a survey on IoT communication environment. They identified some constraints for IoT devices, for example, computing resources and battery lifetime, and some solutions were also suggested. Lyu et al. [43] proposed a “selective authentication based geographic opportunistic routing (SelGOR)” to mitigate the DoS attacks, which can fulfil the requirements of authenticity, integrity and reliability in WSNs. By “statistic state information (SSI)” of links between nodes, SelGOR took the advantage of an SSI-based trust model to increase the network efficiency in terms of data delivery. SelGOR used an entropy-based algorithm to maintain integrity which was also able to detect DoS attacker nodes to improve the performance.

He et al. [44] proposed a scheme called “sector-based random routing (SRR)” to resolve the source location privacy problem. Under the implementation of the proposed method, the energy consumption is also reduced. As per SRR mechanism, the routing paths are disappeared by dividing the network into several sectors, which further improves the security.

Airehrour et al. [45] proposed a SecTrust-RPL protocol that used “SecTrust” system. It identifies some nodes to make decisions for routing using trust. The trust computation was done through exchanged packets between the nodes to determine the trustworthiness. It can identify the attacker nodes and also improve the throughput of the RPL. Sicari et al. [46] proposed a technique to mitigate DoS attack against the IoT middleware, called “networked Smart object (NOS)”. They designed a variable number of dynamic virtual ports on each NOS, and the second thing is that it binds the identifier virtual port of each NOS’s port to UID, which was more tedious to predict by attacker nodes.

Bhosale et al. [47] implemented an intrusion detection technique in which they compared “Received Signal Strength Indicator (RSSI)” value of neighbour nodes and the victim nodes with the threshold values. They kept the record of the broadcasting node’s RSSI value, from where the victim packet was disseminated. It helped to locate the presence of attacker node in their technique. Liu et al. [48] developed a detection mechanism for low rate DOS attack. They used the expired state entries and proposed an “enhanced distributed low-rate attack mitigating (eDLAM)” method to mitigate the attacks. Their presented eDLAM can detect an attack based on expired state-entry numbers.

Raoof et al. [49] presented a comparative analysis of “Routing Protocol for Low-Power and Lossy Networks (RPL)”. Some of the routing attacks along with the mitigation methods were also discussed. Mayzaud et al. [50] presented a method to mitigate version number attacks in RPL networks. This was based on “distributed monitoring architecture” which conserves the energy of resource constrained nodes for the “Advanced Measurement Infrastructures (AMI)”. Their detection procedure is performed by the source node after collecting the detection information from all deployed nodes. In addition, Wazid et al. [16,17,24] also discussed intrusion detection schemes for detection of various attacks, including sinkhole, blackhole, hybrid anomaly as well as routing attacks in hierarchical WSNs and edge-based IoT (EIoT) networks.

In Table 1, the comparison to existing intrusion detection techniques is provided. It contains different parameters such as “name of the technique/protocol”, “its goal”, “method used” and “its outcomes and limitations”.

## 3. The Proposed Sinkhole Attack Detection Scheme

In this section, the different phases of the proposed “sinkhole attack detection scheme for edge-based IoT environment”, called SAD-EIoT, are explained. The different message formats required to describe SAD-EIoT are also presented. Furthermore, the notations and their significance shown in Table 2 are used in the proposed SAD-EIoT.

### 3.1. Network Scenario

For an edge-based IoT environment, Figure 3 suggests that there is a resource-rich edge device (node/router) which works like a gateway node. For instance,“Personal Digital Assistant (PDA)” can be configured as an edge node [51,52,53]. In an edge-based IoT environment, there are resource-constrained IoT smart devices (i.e., IoT sensors) with limited resources (for instance, MICAz motes [53]). The IoT sensors can be deployed randomly or manually in a required area (i.e., in a forest for environment monitoring) based on application scenarios. After deployment, the first task of sensing devices is to find the neighbours in its communication range. To perform such a task, IoT sensors can broadcast “HELLO messages” (containing their identity) to other nodes in their communication ranges. After receiving “HELLO messages” from the neighbouring sensing nodes, each IoT sensor constructs a neighbour list [54]. Every edge node $EN_{j}$ also finds its “physical neighbors” (for example, the IoT sensors). $EN_{j}$ is responsible for anomaly detection in the network. For ensuring secret communications among an edge node and IoT sensing devices, and also among different IoT sensors and itself, a key management protocol, namely the “unconditionally secure deterministic key management” suggested by Das [52] can be utilised. Assume that $SK_{S_{i},S_{j}}$ and $SK_{EN_{j},S_{i}}$ are two different symmetric (secret) keys among two neighbouring IoT sensing nodes, say $S_{i}$ and $S_{j}$ and among an edge node $EN_{j}$ and its neighbour IoT sensing node $S_{i}$. The method for key establishment can be defined on the basis of deterministic key management scheme available in [52]. With the help of the established secret keys, neighbour nodes can securely communicate with each other in the edge-based IoT environment. The delay between $S_{i}$ and $EN_{j}$ can be computed by using the technique given in [17,55].

Assume each transmitted packet of a sensing node contains a distinct sequence number and the sequence numbers are kept in an ascending order. Next, $t_{x}\left(j,k\right)$ denoted as a packet *j*’s receiving time on a node *k* corresponding to the “perfect clock $t_{r}\left(j,k\right)$” and the packet *j*’s transmitting time on the node *k*. The transmission or reception time of a message is considered as the “time just before the first byte of a packet (message) is sent or received”. Let *a* and *b* represent the source and destination nodes along with a chosen path. If $t_{r}\left(j,a\right)$ is another parameter denoting the packet *j*’s generation time on *a*, the packet *j*’s “end-to-end delay for a path” is calculated as [16,17,24]:

$$t_{d}\left(j\right)=t_{r}\left(j,b\right)-t_{r}\left(j,a\right).$$

Now, if the packet *j*’s waiting time at node *k* on the path is represented by $t_{w}\left(j,k\right)$, then $t_{w}\left(j,k\right)=$ $t_{x}\left(j,k\right)-$$t_{r}\left(j,k\right)$. It is important to notice that waiting time $t_{w}\left(j,k\right)$ incorporates node’s backoff time for competing for the channel. Therefore, end-to-end delay is calculated as

$$t_{d}\left(j\right)=\sum_{k=1}^{n-1}t_{w}\left(j,k\right),$$

where the number of total nodes in that path is denoted by *n*. Since the IoT sensors do not have tamper-resistant hardware in general due to the cost factor, an adversary A gets a chance to extract all the required information from the memory of a physically captured IoT sensor node [12,13]. A can then store the extracted information in the memory of the newly manufactured (cloned) IoT sensor node and can also load the sinkhole attack functionality program required to launch that attack in the network.

### 3.2. Process Involved in SAD-EIoT

**Figure 5:**
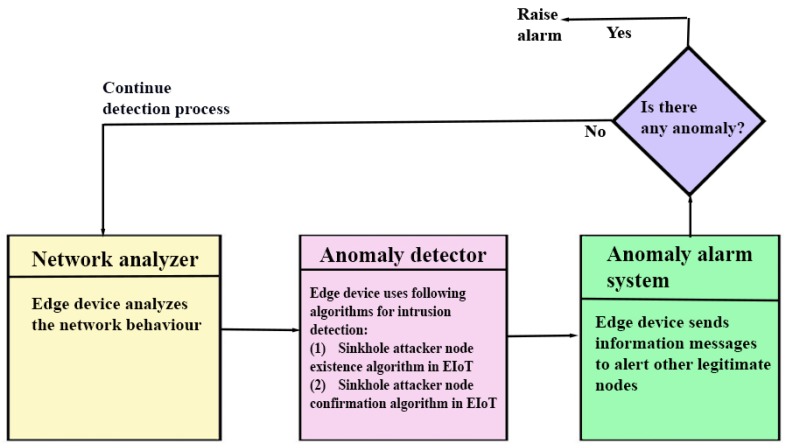
Sequence diagram of sinkhole attack detection through SAD-EIoT.

The process involved in SAD-EIoT is explained using a sequence diagram of sinkhole attack detection through SAD-EIoT (see Figure 5). Its details are given below.

Edge node keeps all the important information, such as identity $\left(ID_{S_{i}}\right)$ of every IoT sensing node $S_{i}$, ranks information and its battery level. As per the nature of sinkhole attack, the malicious node (sinkhole attacker node, say $SHA_{k}$) advertises a shortest path to the destination (i.e., edge node) and the neighbouring IoT sensor nodes get attracted towards that path and send their packets to $SHA_{k}$ as they assume that the shortest path to the destination is through $SHA_{k}$. After receiving the data from neighbour IoT sensors, $SHA_{k}$ can play with the communication. $SHA_{k}$ can perform the following malicious tasks:

- Dropping of the packets
- Modification of information in the packets
- Forwarding the packets selectively (i.e., forwarding of UDP packets and dropping of TCP packets)
- Forwarding the packets with some delay

The sinkhole attack can disturb the overall configuration of the network as it affects most of the important network parameters. For instance, it may reduce the throughput (sometimes, it tends to zero when $SHA_{k}$ drops all packets) and increase the end-to-end delay along with extremely low packet delivery ratio [16,17,22,24]. Since an edge node is resource rich in the network, it can be easily used for detection of sinkhole attacks. The detection of sinkhole attack is executed in the following two phases:

**Phase 1 (Identifying the presence of sinkhole attacker nodes):** In this phase, we identify the existence of SHAs in the network by applying the steps in Algorithm 1. The parameters, such as node identity $ID_{S_{i}}$, hop count from $EN_{j}$ ($HC_{S_{i}}$), remaining energy at the nodes $REN_{S_{i}}$ and rank information $R_{S_{i}}$ are used. An IoT node $S_{i}$ is recognised as a suspected SHA, if following conditions hold [9,17,18]:

- $HC_{S_{i}}<HC_{\theta }$
- $REN_{S_{i}}<REN_{S_{i_{\theta }}}$
- $R_{S_{i}}\notin \left\{R_{LS_{i}},R_{U_{S_{i}}}\right\}$

Here, $HC_{\theta }$ and $REN_{S_{i_{\theta }}}$ are threshold values of the network hop count and remaining energy, respectively. $R_{LS_{i}}$ and $R_{U_{S_{i}}}$ are lower and upper limits of ranks for a particular node (i.e., $S_{i}$), respectively. A node loses some energy whenever it transmits or receives packets and the edge node knows about the initial battery status of all the nodes. If an attacker node provides modified battery status to the edge node, in turn that edge node can calculate the battery (energy) value using the available technique in the literature. A similar approach can be applicable for hop counts and rank information. By the end of this phase, a list ${⋃}_{l}$ of suspected attacker nodes is prepared, if they exist in the network.

**Phase 2 (Confirming the existence of sinkhole attacker nodes):** In this phase, the confirmation of identified nodes as the sinkhole attacker or some battery drained nodes is done. The steps stated in Algorithm 2 are required to perform this task. In this phase of attack detection, the possible cases are outlined below.

- **Case 1:** If $EN_{j}$ does not get messages from a doubtful node $S_{i}$, it attempts to figure out that node $S_{i}$ as $SHA_{k}$ or normal (genuine) node which has depleted its entire battery (might be a node failure). In such a situation, $EN_{j}$ transmits $\mu_{sdq}$ to $S_{i}$ and waits some time for its response. If the condition $WT>WT_{\theta }$ holds, where $WT_{\theta }$ is the waiting time’s threshold, it will indicate the expiry of waiting time. If both response message $\left(\mu_{sr}\right)$ as well as data message $\left(\mu_{d}\right)$ from $S_{i}$ are not received by $EN_{j}$, it makes a decision as the $S_{i}$’s failure. Note that additional factors like network congestion have been included in $WT_{\theta }$.
- **Case 2:** If $EN_{j}$ gets $\mu_{sr}$, but the $\mu_{d}$ is not received by $EN_{j}$, $S_{i}$ is identified as the “sinkhole attacker node” $SHA_{k}$. It is also determined that $SHA_{k}$ is a kind of SHA which consumes all packets and does not forward them towards the destination (i.e., $EN_{j}$).
- **Case 3:** If $EN_{j}$ receives the $\mu_{sr}$ and also $\left(\mu_{d}\right)$ from $S_{i}$, it checks the integrity of $\mu_{d}$ by using hashing algorithm (i.e., SHA-1 or SHA-256 [56]). If the integrity does not hold, $S_{i}$ is treated as the $SHA_{k}$ which has modified $\mu_{d}$.
- **Case 4:**$EN_{j}$ receives $\mu_{sr}$ and also $\mu_{d}$ from $S_{i}$, but the quality of service of the network is not up to the mark [57]. Since $EN_{j}$ is a powerful node, it can run some technique to maintain the quality of the service of the network [57]. For example, $SHA_{k}$ may forward the UDP packets but not the TCP packets. If all these features are included then it becomes a sign of selective forwarding of packets (a kind of packet forwarding attack) [57,58,59]. For the detection purpose, $EN_{j}$ can execute the following steps if the count of packets for a particular service (i.e., TCP) does not exceed the threshold value of count of packets in a particular duration of time. EN considers $S_{i}$ node as the $SHA_{k}$. Further, note that the threshold value of count of packets in a particular duration of time is an empirical value which can be set at the EN at the time of the deployment of the nodes in the network.
- **Case 5:** When the $\mu_{sdq}$ is transmitted by $EN_{j}$ to $S_{i}$, it waits some time for the response message. If the condition $WT>WT_{\theta }$ holds, it will indicate the expiry of waiting time. If $EN_{j}$ receives $\mu_{sr}$, it waits for receipt of $\mu_{d}$ from $S_{i}$. If $EN_{j}$ receives $\mu_{d}$ after the expiry of the waiting time (i.e., $WT>WT_{\theta }$), the node $S_{i}$ is detected as the $SHA_{k}$ because it delays the packets before forwarding them towards the destination (i.e., $EN_{j}$).

As the detection work proceeds with the time, EN tries to detect malicious sinkhole (attacker) nodes and also adds them to the list of sinkhole attacker nodes $SHA_{list}$. In the “anomaly alarm system phase”, $EN_{j}$ ignores the detected $SHA_{k_{i}}$, where i=1, 2, …, *n* and raises an alert to warn the other legitimate nodes about the existence of SHAs. After that, the legitimate IoT devices remove the entry of $SHA_{k}$ node from its neighbour list and start sending their packets to the other possible route(s).

The sequence diagram of sinkhole attack detection through SAD-EIoT provided in Figure 5 is helpful to explain the overall communication process of SAD-EIoT. It has the following important stages:

- **Network analyser:** An edge node $EN_{j}$ performs the analysis of the network behaviour. $EN_{j}$ then identifies normal and abnormal activities of the network.
- **Anomaly detector:** For the detection of SHAs, two different phases are used, namely *Phase 1* for identifying the existence of SHAs and *Phase 2* for confirming the existence of SHAs. $EN_{j}$ does the work of sinkhole node detection by using the steps of SHA existence algorithm in EIoT (Algorithm 1). After the completion of all steps mentioned in Phase 1, a list of doubted nodes ${⋃}_{l}$ is constructed that may or may not have the attacker nodes. To confirm the existence of SHAs in the network, $EN_{j}$ executes the steps of SHA confirmation algorithm in EIoT (see Algorithm 2). After the successful completion of Phase 2, a list of confirmed SHAs and $SHA_{list}$ is obtained which contains the entries for all types of SHAs which exist in the network.
- **Alarm system:** After the successful completion of both phases of SHAs detection, the list $SHA_{list}$ is generated. The $EN_{j}$ blacklists these malicious nodes and also sends alarm messages to other legitimate IoT devices (i.e., IoT sensor nodes). Then, these legitimate nodes remove the entries of SHAs from their neighbour list and start sending their packets to the other possible available route(s).

### 3.3. Formats of Messages Used in Sinkhole Attack Detection

SAD-EIoI uses four messages which are also utilised in some existing techniques: [16,17,18,24], namely, (i) “status and data query message $\mu_{sdq}$”, (ii) “status response message $\mu_{sr}$”, (iii) “data message $\mu_{d}$” and (iv) “information message $\mu_{in}$”. The structures of these different messages are provided below.

**Figure 6:**
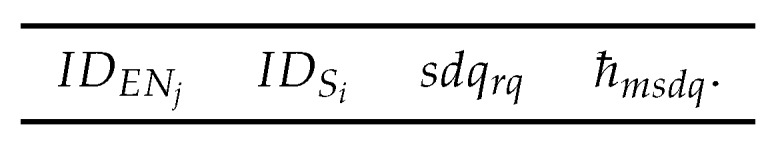
Assembly of status and data query message $\left(\mu_{sdq}\right)$.

**Figure 7:**
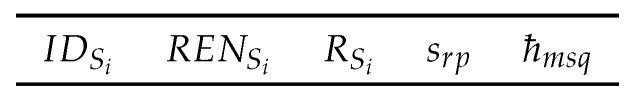
Structure of status response message $\mu_{sr}$.

**Figure 8:**
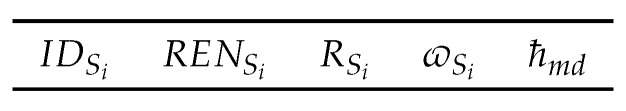
Assembly of data message $\mu_{d}$.

**Figure 9:**
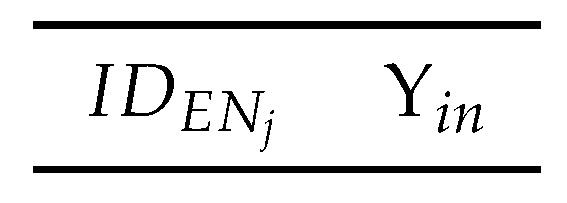
Structure of information message $\left(\mu_{in}\right)$.

- *Status and data query message*$\mu_{sdq}$: The message $\mu_{sdq}$ is shown in Figure 6. $EN_{j}$ transmits $\mu_{sdq}$ to all IoT devices (sensors). This message is constructed using the different fields, such as an $EN_{j}$’s identity $ID_{EN_{j}}$, an IoT device $S_{i}$’s identity $ID_{S_{i}}$, the information field $sdq_{rq}$ and also the hashed message authentication code $\left(\hbar_{msdq}\right)$, where $\hbar_{msdq}=$ $h(SK_{EN_{j},S_{i}}$$||ID_{EN_{j}}$$||ID_{S_{i}}$$||sdq_{rq})$.
- *Status response message*$\left(\mu_{sr}\right)$: The structure of $\mu_{sr}$ provided in Figure 7 is composed of different fields, such as $ID_{S_{i}}$, “remaining energy (battery power)” $REN_{S_{i}}$ of $S_{i}$, “rank information” $R_{S_{i}}$ of $S_{i}$, the information field $s_{rp}$ and $\hbar_{msq}=$ $h(SK_{EN_{j},S_{i}}||$$ID_{S_{i}}||REN_{S_{i}}\left|\right|$$R_{S_{i}}\left|\right|s_{rp})$. $S_{i}$ sends the message $\mu_{sr}$ to $EN_{j}$. For saving energy, an IoT sensing device can utilize any one of the modes (“sleep”, “idle” and “working”) [60,61]. For the detection of sinkhole attack, the information about the two modes is needed (i.e., “idle” and “working”), because sensing devices cannot respond when they are in the sleeping state. The $s_{rp}$ may contain two response types: 0 (idle state) and 1 (working state).
- *Data message*$\left(\mu_{d}\right)$: The structure of $\mu_{d}$ provided in Figure 8 is composed of different fields, for example, $ID_{S_{i}}$, $REN_{S_{i}}$ and $R_{S_{i}}$ of $S_{i}$, $\varpi_{S_{i}}$ as the sensing data needs to be transmitted to EN, and $\hbar_{md}$$=h(SK_{EN_{j},S_{i}}$$||ID_{S_{i}}$$||REN_{S_{i}}\left|\right|R_{S_{i}}\left||\varpi \right)$. Note that session key $SK_{EN_{j},S_{i}}$ can be used to encrypt the data, if it is required.
- *Information message*$(\mu_{in}):$ After performing the detection of SHAs, $EN_{j}$ sends the information message to alert the other legitimate IoT sensors. The structure of $\mu_{in}$ provided in Figure 9 is also composed of different fields, like $ID_{EN_{j}}$ and detection information field $\Upsilon_{in}$ contains the information of the detected SHAs.

### 3.4. Research Methodology of SAD-EIoT

In this section, the research methodology of SAD-EIoT is discussed. SAD-EIoT can detect the sinkhole attacker nodes in an EIoT environment efficiently. The detection procedure happens in two phases. Phase 1 identifies the SHAs by the “sinkhole attacker node existence algorithm”, whereas Phase 2 allows to see the existence of the doubted nodes (either normal or SHAs) are identified by using the “sinkhole attacker node confirmation algorithm”. These phases are discussed in the subsequent sections.

#### 3.4.1. Sinkhole Attacker Node Existence Algorithm in EIoT

The “sinkhole attacker node existence algorithm in EIoT” is discussed in Algorithm 1 which is used to recognise the existence of the suspected SHAs. It utilises various parameters, such as node $S_{i}$’s identity $ID_{S_{i}}$, hop count $HC_{S_{i}}$ from $EN_{j}$, remaining energy at nodes $REN_{S_{i}}$ and rank information $R_{S_{i}}$. This algorithm finds the SHAs, in the case of a sensor node $S_{i}$, if the conditions $HC_{S_{i}}<HC_{\theta }$, $REN_{S_{i}}<REN_{S_{i_{\theta }}}$ and $R_{S_{i}}\notin \left\{R_{LS_{i}},R_{U_{S_{i}}}\right\}$ are satisfied, where $HC_{S_{i_{\theta }}}$ and $REN_{S_{i_{\theta }}}$ are threshold values of hop count and remaining energy, respectively. Moreover, $(R_{LS_{i}}$, $R_{U_{S_{i}}})$ is a pair of lower and upper limits of rank for a particular node $S_{i}$. Algorithm 1 also provides a list of suspicious attacker nodes, say ${⋃}_{l}$, if these suspicious SHAs exist in the network.

**Remark** **1.** *If the “hop count” $HC_{S_{i}}$ of an IoT sensing node $S_{i}$ from $EN_{j}$ is less than the network hop count threshold $HC_{\theta }$, that is, if $HC_{S_{i}}<HC_{\theta }$, $S_{i}$ may be considered as SHA.*

**Example** **1.** *To validate the statement of Remark 1, the scenario provided in Figure 4 should be considered. If a node $S_{i}$ is far away from a destination $EN_{j}$, that is, its hop count value from $EN_{j}$ is high, chances are that this will not be an exact SHA. In order to be an exact SHA, this particular node should be as close as possible to the destination $EN_{j}$. If a sinkhole attacker node is closer to $EN_{j}$, it can get a greater number of packets from the neighbour nodes and it may further damage the network operations quickly. Otherwise, if the SHA is far away from the destination $EN_{j}$, in that case it will not get a greater number of packets. In this situation, the damage to the network will be minimum. Thus, an attacker’s advantage to launch the sinkhole attack will be very low. The empirical threshold value of hop count of the network, $HC_{\theta }$, can be set at the time of the deployment of the IoT sensors and it can be compared to the hop count value for that particular node $HC_{S_{i}}$ to identify a sign of intrusion in the network. Therefore, if $HC_{S_{i}}<HC_{\theta }$ holds, $S_{i}$ can be treated as a suspected SHA.*

**Remark** **2.** *If the “remaining energy under the normal behaviour and abnormal behaviour of an IoT sensing node $S_{i}$” are $REN_{S_{i_{\theta }}}$ and $REN_{S_{i}}$, respectively, the criteria $REN_{S_{i}}$$<REN_{S_{i_{\theta }}}$ needs to hold for a sinkhole attack.*

**Example** **2.** *To justify Remark 2, the scenario available in Figure 4 should be considered. A node $S_{i}$ receives a lower number of messages if it is a normal node. However, if it is a SHA, it will definitely receive a greater number of messages as per the mechanism of a sinkhole attack. When a node receives a greater number of packets, its battery depletion will be greater as compared to the normal node. Suppose $EN_{j}$ sets a threshold value of remaining energy for a particular node $S_{i}$ as $REN_{S_{i_{\theta }}}$ and in actual scenario it is $REN_{S_{i}}$. So, if a node $S_{i}$ is a SHA, the condition $REN_{S_{i}}$$<REN_{S_{i_{\theta }}}$ turns out to be valid. Otherwise, $S_{i}$ is a normal sensor node of EIoT. Therefore, in the case of sinkhole attack, the condition $REN_{S_{i}}<$$REN_{S_{i_{\theta }}}$ becomes true.*

**Algorithm 1** Sinkhole attacker node existence algorithm in EIoT.
1:**for** each edge node $EN_{j}$ in edge-based IoT environment **do**2: $EN_{j}$ sends status and data query message $\left(\mu_{sdq}\right)$ to the IoT sensors, $S_{i}$.3: After receiving $\mu_{sdq}$, each $S_{i}$ computes $\hbar_{msdq}^{\prime }=$$h(SK_{EN_{j},S_{i}}$$||ID_{EN_{j}}$$||ID_{S_{i}}$$||sdq_{rq})$ using the shared secret key $SK_{EN_{j},S_{i}}$ with $EN_{j}$.4: **if** $\left(\hbar_{msdq}^{\prime }=\hbar_{msdq}\right)$ **then**5:  $\mu_{sdq}$ is valid and $S_{i}$ responses with status response message $\mu_{sr}$ $=\langle ID_{S_{i}},REN_{S_{i}},$ $R_{S_{i}},s_{rp},$ $\hbar_{msq}\rangle$ to $EN_{j}$ using its “current remaining energy” $REN_{S_{i}}$ and “rank information” $R_{S_{i}}$.6:  After receiving $\mu_{sr}$, $EN_{j}$ recomputes $\hbar_{msq}^{\prime }=$ $h(SK_{EN_{j},S_{i}}||$$ID_{S_{i}}||REN_{S_{i}}\left|\right|$$R_{S_{i}}\left|\right|s_{rp})$ using the “shared secret key $SK_{EN_{j},S_{i}}$” with $S_{i}$.7:  **if** $(\hbar_{msq}^{\prime }=$ $\hbar_{msq})$ **then**8:   $\mu_{sr}$ is a genuine message.9:  **end if**10: **end if**11: Each $S_{i}$ in edge-based IoT environment sends message $\mu_{d}$ $=\langle ID_{S_{i}},$ $REN_{S_{i}},R_{S_{i}},$ $\varpi ,\hbar_{md}\rangle$, if it has anything to send, to $EN_{j}$ using its “current remaining energy” $REN_{S_{i}}$ and “rank information” $R_{S_{i}}$.12: After receiving $\mu_{d}$ from $S_{i}$, $EN_{j}$ recomputes $\hbar_{md}^{\prime }$ $=h(SK_{EN_{j},S_{i}}$$||ID_{S_{i}}$$||REN_{S_{i}}\left|\right|R_{S_{i}}\left|\right|\varpi_{S_{i}})$ using the “shared secret key $SK_{EN_{j},S_{i}}$” with $S_{i}$.13: **if** $(\hbar_{md}^{\prime }=$ $\hbar_{md})$ **then**14:  $\mu_{md}$ is valid.15: **end if**16: Based on information gathered by IoT sensor $S_{i}$, $EN_{j}$ checks following condition.17: **if** (($HC_{S_{i}}<HC_{\theta }$) & ($REN_{S_{i}}<REN_{S_{i_{\theta }}}$) & ($R_{S_{i}}\notin \left\{R_{LS_{i}},R_{U_{S_{i}}}\right\})$) **then**18:  Node $S_{i}$ is considered as a suspected SHA.19:  Add $S_{i}$ in ${⋃}_{l}$.20:  Execute sinkhole attacker node confirmation algorithm provided in Algorithm 2.21: **end if**22:
**end for**



#### 3.4.2. Sinkhole Attacker Node Confirmation Algorithm in EIoT

The “sinkhole attacker node existence algorithm in EIoT” provides a list of suspected attacker nodes. However, to prove a suspected node is an attacker node, the sinkhole attacker node confirmation algorithm in EIoT, which comes under Algorithm 2, is required.

To perform this task, $EN_{j}$ executes the following steps. If an edge node $EN_{j}$ does not receive data packets from a suspicious node $S_{i}$, it will try to segregate that node $S_{i}$ as $SHA_{k}$ or normal (genuine) node which completely drained its battery (due to node malfunctioning). In such case, $EN_{j}$ sends $\mu_{sdq}$ to $S_{i}$ and waits for some time for its response. If $WT>WT_{\theta }$ holds, it indicates the expiry of waiting time. If $EN_{j}$ does not get $\mu_{sr}$ and also $\mu_{d}$ from $S_{i}$, it is the case of failure of $S_{i}$. Note that in the threshold value of waiting time other factors, such as network congestion, are also included. If $EN_{j}$ receives $\mu_{sr}$, but not $\mu_{d}$, $S_{i}$ is confirmed as $SHA_{k}$. It is also determined that $SHA_{k}$ is a kind of SHA which consumes all packets and does not forward them towards the destination ($EN_{j}$). If $EN_{j}$ receives the $\mu_{sr}$ and also $\mu_{d}$ from $S_{i}$, $EN_{j}$ checks the integrity of $\mu_{d}$ by using hash algorithm (i.e., SHA1 or SAH256) [56]. If the integrity does not hold, $S_{i}$ is treated as the $SHA_{k}$ which can modify $\mu_{d}$. Another case is that $EN_{j}$ receives $\mu_{sr}$ and also $\mu_{d}$ from $S_{i}$ but the quality of service of the network is not up to the mark [57]. $EN_{j}$ is a powerful node which can keep the quality of the communication up to the mark [57]. For example, $SHA_{k}$ may forward the UDP packets but not the TCP packets. If all these features are included then it becomes a sign of selective forwarding of packets (a kind of packet forwarding attack) [57,58,59]. For the detection purpose, $EN_{j}$ can execute the following steps if the count of packets for a particular service (i.e., TCP) is less than the threshold value of count of packets in a particular duration of time, that is, $PC_{S_{i}}<PC_{S_{i}{}_{\theta }}$. Then $EN_{j}$ considers $S_{i}$ node as $SHA_{k}$. Further note that the threshold value of count of packets in a particular duration of time is an empirical value which can be set at $EN_{j}$ at the time of the deployment of the different nodes. When $EN_{j}$ transmits the $\mu_{sdq}$ to $S_{i}$, it waits for some time for its response. If $WT>WT_{\theta }$ holds, where $WT_{\theta }$ is the “threshold value of the waiting time”, it indicates the expiry of waiting time. If $EN_{j}$ receives $\mu_{sr}$, it waits for the receiving of $\mu_{d}$ from $S_{i}$. If $EN_{j}$ receives $\mu_{d}$ after the expiry of the waiting time ($WT>WT_{\theta }$), the node $S_{i}$ is identified as $SHA_{k}$ which can delay the packets before forwarding them towards the destination ($EN_{j}$). After the detection, $EN_{j}$ blacklists and adds SHAs to the list $SHA_{list}$.
**Algorithm 2** Sinkhole attacker node confirmation algorithm in EIoT1:**for** each edge node $EN_{j}$ in edge-based IoT environment **do**2: **if** edge node $EN_{j}$ does not receive any message **then**3:  $EN_{j}$ transmits $\mu_{sdq}$ to a node $S_{i}$.4:  Set WT as WT=WT+1.5:  **if** $WT>WT_{\theta }$ **then**6:   **if** $EN_{j}$ receives $\mu_{sr}$ but not $\mu_{d}$ from node $S_{i}$ **then**7:    $S_{i}$ considered to sinkhole attacker node $SHA_{k}$ which drops packets.8:   **else if** $EN_{j}$ receives both $\mu_{sr}$ & $\mu_{d}$ from node $S_{i}$ and $\hbar_{md}^{\prime }$$\neq \hbar_{md}$ **then**9:    $S_{i}$ considered to be $SHA_{k}$ which modifies the packets.10:   **else if** $EN_{j}$ receives both $\mu_{sr}$ & $\mu_{d}$ from node $S_{i}$ and $PC_{S_{i}}<PC_{S_{i}{}_{\theta }}$ **then**11:    $S_{i}$ considered to be $SHA_{k}$ which selectively forwards the packets.12:   **else if** $EN_{j}$ receives both $\mu_{sr}$ & $\mu_{d}$ from node $S_{i}$ and $WT>WT_{\theta }$ **then**13:    $S_{i}$ considered to be $SHA_{k}$ which delays packets before forwarding them.14:   **else**15:    Edge node $EN_{j}$ does not receive the messages $\mu_{sr}$ and $\mu_{d}$ from the node $S_{i}$.16:    Failure of a node is detected.17:   **end if**18:  **end if**19:  $EN_{j}$ blacklists the detected nodes and adds its entry in the list $SHA_{list}$, and broadcasts its identity $ID_{SHA_{k}}$ to all legitimate IoT sensors.20: **end if**21:**end for**

## 4. Mathematical Models for SAD-EIoT

In this part of the paper, the various mathematical models utilised in SAD-EIoT such as packet delivery ratio, “throughput” and end-to-end delay for edge-based IoT environment are explained [16,17,24].

### 4.1. Packet Delivery Ratio

Suppose the symbols $PD_{n}$, $PD_{a}$ and $PD_{s}$ are respectively the “packet delivery ratios” associated with the normal flow, sinkhole attack and SAD-EIoT. Further, assume that $|\mu_{d}|$, $|\mu_{d^{\prime }}|$, $|\mu_{dpa}|$, $|\mu_{dpa^{\prime }}|$ and $|\mu_{d_{1}}|$ represent the count of “data packets” sent by IoT sensing devices, “authentic packets” received by an edge node, $|\mu_{dpa}|$, “data packets that are not transmitted by sinkhole nodes”, “data packets that are not transmitted by the sinkhole nodes (TP)” and “data packets that are not transmitted by sinkhole nodes (FN)”, respectively. Thus it is clear that $|\mu_{d_{1}}|$ = $|\mu_{dpa}|-$$|\mu_{dpa^{\prime }}|$. The estimation of “packet delivery ratio (PD)” with respect to “normal traffic flow” is given as [16,17,24]:

$$PD_{n}=\frac{|\mu_{d^{\prime }}|}{|\mu_{d}|}.$$

Under sinkhole attack, PD can be computed as

$$PD_{a}=\frac{|\mu_{d^{\prime }}\left|-\right|\mu_{dpa}|}{|\mu_{d}|}.$$

PD under the proposed method “SAD-EIoT” can also be formulated as

$$PD_{s}=\frac{|\mu_{d^{\prime }}\left|-\right|\mu_{d_{1}}|}{|\mu_{d}|}.$$

The packet loss rate is an additional important network parameter that is explained as the number of lost packets per unit time and it can be computed as $\frac{\nu_{lp}}{T_{d}}$ where the “total time (in seconds)” is $T_{d}$ and the total lost packets is denoted by $\nu_{lp}$. It is also very important for a dependable network communication to keep packet loss rate as low as possible. The mathematical model for packet loss rate can be defined in a similar way as the packet delivery ratio.

### 4.2. Throughput

Let $\Lambda_{n}$, $\Lambda_{a}$ and $\Lambda_{s}$ represent the “throughput of the network” under the various scenarios, for example, “normal flow”, sinkhole attack and SAD-EIoT, respectively. Let us assume $T_{n}$, $T_{a}$ and $T_{s}$ are the “packets delivery time” under “normal flow”, sinkhole attack and SAD-EIoT, respectively. Then, the throughput under normal flow of traffic as follows [16,17,24]:

$$\Lambda_{n}=\frac{|\mu_{d^{\prime }}|\times pkt_{s}}{T_{n}}.$$

Similarly, the throughput under sinkhole attack can be computed as

$$\Lambda_{a}=\frac{pkt_{s}\times \left(\right|\mu_{d^{\prime }}\left|-\right|\mu_{dpa}\left|\right)}{T_{a}},$$

and the throughput under the deployment of SAD-EIoT is represented by

$$\Lambda_{s}=\frac{pkt_{s}\times \left(\right|\mu_{d^{\prime }}\left|-\right|\mu_{d_{1}}\left|\right)}{T_{s}},$$

where a data packet size is represented by $pkt_{s}$.

### 4.3. End-to-End Delay

Let $\nu_{n}$, $\nu_{a}$ and $\nu_{s}$ be the “end-to-end delays” under different scenarios such as “normal flow”, sinkhole attack and SAD-EIoT, respectively. Then, the end-to-end delay under normal flow of traffic can be approximated as [16,17,24]:

$$\nu_{n}=\nu ,$$

where ν can be represented as

$$\nu =\frac{\sum_{i=1}^{p}\left(T_{rec_{i}}-T_{send_{i}}\right)}{p},$$

$T_{rec_{i}}$ is the “receiving time”, $T_{send_{i}}$ is the “sending time” of a “packet *i*” and *p* is the “total number of packets”.

The end-to-end delay under sinkhole attack is approximated as

$$\nu_{a}=\nu_{n^{\prime }}+\nu_{n_{sha}},$$

where *n* denotes total IoT sensing nodes in the network, $n_{sha}$ is the count of sinkhole nodes, $n^{\prime }=$$n-n_{sha}$ counts towards “number of normal nodes for sinkhole attack scenario” and $\nu_{n_{sha}}$ is the “delay corresponding to $n_{sha}$ sinkhole attacker nodes”. Finally, the “end-to-end delay under the proposed SAD-EIoT” can be computed as

$$\nu_{s}=\nu_{n^{{}^{\prime \prime }}}+\nu_{FN_{ra}},$$

where $FN_{ra}$ is the “number of nodes identified as normal nodes” by SAD-EIoT, but these are actually sinkhole nodes, $n^{{}^{\prime \prime }}=$$n-n_{FN_{sha}}$ is the normal node count in SAD-EIoT and $\nu_{FN_{sha}}$ represents the delay associated with $FN_{sha}$ nodes. Hence, if the count of false negative nodes is zero, the end-to-end delay will be $\nu_{s}=\nu_{n}$.

## 5. Analysis of SAD-EIoT

In this part of the paper, the analysis of the security of SAD-EIoT, along with its communication and computational costs is conducted.

### 5.1. Security Analysis

For an IoT sensing node, say $S_{i}$, the corresponding edge node has the responsibility to keep information like its identity $\left(ID_{S_{i}}\right)$, its “remaining energy $REN_{S_{i}}$”, hop count value $HC_{S_{i}}$ and “rank information $R_{S_{i}}$”. If a SHA is placed successfully in EIoT then it can start to damage normal network operations (i.e., transmitted packets can be dropped, delayed, updated or selectively forwarded). Designed SAD-EIoT has the capability to detect SHAs. This work is divided into two phases. In “phase 1”, it first identifies the existence of suspected SHAs in EIoT by using the steps of “Sinkhole attacker node existence algorithm in EIoT (Algorithm 1)”. This algorithm uses parameters, such as node $S_{i}$’s identity $\left(ID_{S_{i}}\right)$, its “remaining energy $REN_{S_{i}}$”, hop count value $HC_{S_{i}}$ and “rank information $R_{S_{i}}$”. A node $S_{i}$ is identified as a suspected SHA if $HC_{S_{i}}<HC_{\theta }$, $REN_{S_{i}}<REN_{S_{i_{\theta }}}$ and $R_{S_{i}}\notin \{R_{LS_{i}},$$R_{U_{S_{i}}}\}$ where $HC_{\theta }$ and $REN_{S_{i_{\theta }}}$ are threshold values of network hop count, remaining energy and $R_{LS_{i}}$, $R_{U_{S_{i}}}$ are lower and upper limits of ranks for a particular node (i.e., $S_{i}$). After successful execution of phase 1, the “sinkhole attacker node confirmation algorithm in EIoT (Algorithm 2)” is accomplished in phase 2. If an edge node $EN_{j}$ does not receive messages from a particular node $S_{i}$, then first it confirms the node $S_{i}$ is SHA or “a case of node failure”. To confirm this $EN_{j}$ sends the messages $\mu_{sdq}$ to node $S_{i}$, and starts the “waiting time counter”. If waiting time is over and $EN_{j}$ does not receive $\mu_{sr}$ and $\mu_{d}$ from IoT sensor $S_{i}$, then it is determined that this is a case of node failure (i.e., $S_{i}$ is a failure node). Otherwise, if $EN_{j}$ receives the $\mu_{sr}$, but it does not receive $\mu_{d}$, $S_{i}$ is detected as SHA which has the capability to drop the packets. All these analyses are preformed by using the above two cases discussed in Section 3.4.2. Similarly, the security of SAD-EIoT for other types of SHAs can be confirmed. Therefore, designed SAD-EIoT is capable enough to defend edge-based IoT communication environment from different types of SHAs.

### 5.2. Communication Cost

For the communication cost analysis, *n* nodes in edge-based IoT environment are considered. In a scenario of a normal flow of traffic, each edge node $EN_{j}$ sends *n* number of messages $\mu_{sdq}$” to IoT sensors. Then IoT sensors have to reply with *n* number of “status response messages” to $EN_{j}$. Moreover, $EN_{j}$ also receives at most *n* number of “data messages $\mu_{d}$”. Therefore, total number of messages exchanged in the case of normal flow of traffic can be estimated as 3n. Whereas in the case of sinkhole attack, $EN_{j}$ only gets $n+(n_{sad}-n_{ssf})$ “data messages $\mu_{d}$” where $n_{sad}$ are the messages dropped by sinkhole attacker nodes (SHAs) which drop the packets and $n_{ssf}$ are the messages dropped by SHAs which selectively drop (in selective forwarding case) the packets. The total number of different messages exchanged under sinkhole attack can be estimated as $(n+n+\left(n+\left(n_{sad}-n_{ssf}\right)\right))=$$3n-n_{sad}-n_{ssf}$.

Under the scenario of SAD-EIoT, when $EN_{j}$ does not receive the data messages from some of the SHAs, it resends $n_{sad}+n_{ssf}$ number of $\mu_{sdq}$ messages only to sinkhole message dropping attacker nodes and sinkhole selective forwarding attacker nodes. The sinkhole message dropping attacker and sinkhole selective forwarding attacker nodes send only $\mu_{sr}$ messages but not $\mu_{d}$ messages. Note that $EN_{j}$ receives $n_{sadl}$ and $n_{smd}$ data messages from SHAs which delay the packets and SHAs which modify the packets. Whereas $EN_{j}$ only receives $n_{sad}+n_{ssf}$ number of $\mu_{sr}$ messages. After the successful completion of both phases of proposed mechanism, $EN_{j}$ identifies the different types of sinkhole nodes and sends $n-(n_{sadl}+n_{smd}+n_{sad}+n_{ssf})$ information messages to alert the other legitimate nodes of the network. Where $n_{sadl}$ are messages corresponding to SHAs which delay the packets and $n_{smd}$ are SHAs which modify the packets. It is understood that $EN_{j}$ does not transmit any information messages to SHAs. Hence, as a result, the total number of messages exchanged under the implementation of SAD-EIoT can be estimated as $\left[n+n+\left(n_{sadl}+n_{smd}\right)+\left(n_{sad}+n_{ssf}\right)+\left(n_{sad}+n_{ssf}\right)+\left(n-\left(n_{sadl}+n_{smd}+n_{sad}+n_{ssf}\right)\right)\right]$$=3n+n_{sad}+n_{ssf}$.

It is assumed that identity, “hash digest (output) (if we apply SHA-1 hash algorithm)”, “remaining energy field”, “rank information field” and data fields in various types of messages are of 32 bits, 160 bits, 32 bits, 32 bits and 160 bits, respectively. Therefore, different messages’ sizes can be estimated as $\mu_{sdq}$, $\mu_{sr}$, $\mu_{d}$ and $\mu_{in}$ require 384 bits, 416 bits, 416 bits and 192 bits, respectively.

### 5.3. Computation Cost

As discussed earlier, SAD-EIoT is divided into two phases. In the first phase of SAD-EIoT, the presence of suspected attacker nodes are detected using the steps of the “sinkhole attacker node existence algorithm in EIoT”. Further note that these nodes may or may not be the attacker nodes. If SHAs exist in EIoT, this will be confirmed by Algorithm 2. First Algorithm 1 is executed and then Algorithm 2 will be executed. The different steps of Algorithm 1 and Algorithm 2 are executed in linear time, which can be executed with time complexity O(n), where *n* is number of IoT sensing nodes installed in EIoT. Thus, the cumulative time complexity of SAD-EIoT is estimated as O(n) which is needed for an $EN_{j}$.

**Remark** **3.** *It is important to notice that in designed SAD-EIoT, an IoT sensor node $S_{i}$ needs to send one “status response message $\mu_{sr}$” and one $\mu_{d}$ to an edge node $EN_{j}$. Furthermore, $S_{i}$ needs to compute “two HMAC operations” in the transmission of $\mu_{sr}$ and $\mu_{d}$ messages. Apart from that $S_{i}$ needs another HMAC operation in the validation of $\mu_{sdq}$ message. Hash function computations are very lightweight which again surges in very low computational cost for $S_{i}$ node. The provided estimation infers that SAD-EIoT is very helpful and handy for the “extremely resource-constrained IoT sensors in EIoT” due to low computational cost and lower number of messages exchanged.*

## 6. Practical Implementation of SAD-EIoT

In this part of the paper, the designed SAD-EIoT is practically implemented using the widely-used NS2 2.35 simulation software tool [62].

### 6.1. Simulation Environment

SAD-EIoT is implemented on Ubuntu Linux 14.04 LTS platform using the NS2 simulation software tool [16,17,18]. The considered deployment area is 650 × 250 m${}^{2}$. In the considered deployment field, 121 nodes which consist of different devices such as cloud server, IoT sensor nodes and edge router are placed. This deployment field consists of one cloud server along with six edge nodes. Table 3 consists of values of different simulation parameters used in the practical demonstrations. The “Constant bitrate (CBR)/UDP (User Datagram Protocol)” is treated as the traffic type. The considered routing protocol is “Ad Hoc On-Demand Distance Vector (AODV)” which is applicable for routing methods in wireless communications. That supports both “unicast as well as multicast routing” mechanism [63]. The communication range of an IoT device (sensor) is taken to be 100 m.

### 6.2. Simulation Scenarios

The simulations of EIoT environment are performed for different cases such as normal traffic flow, under sinkhole attack and under the deployment of SAD-EIoT. The information about the different scenarios is provided below.

- **Scenario of normal flow of traffic:** The scenario of EIoT in the case of normal flow of traffic is simulated, containing all 121 normal nodes. Therefore, traffic of the network flows normally without any problem.
- **Scenario of sinkhole attack:** The scenario of EIoT under sinkhole attack is further simulated which consists of 20% attacker nodes i.e., 24 IoT sensor nodes becomes SHAs. Remaining nodes are normal nodes out of 121. The 24 attacker nodes contain various types of nodes like the “sinkhole attacker nodes which drop the packets”, “sinkhole nodes which delay the messages”, “sinkhole nodes which modify the messages” and “sinkhole nodes which selectively forward the messages”.
- **Scenario of SAD-EIoT:** The EIoT scenario under sinkhole attack along with the implementation of SAD-EIoT is further simulated. For the detection of SHAs, each edge node transmits and receives various types of messages in EIoT. After performing the detection process $EN_{j}$ blacklists all detected SHAs and also informs other legitimate IoT sensor nodes through alert (information) messages.

### 6.3. Discussion on Simulation Results

In this part of the paper, the following statistics for SAD-EIoT are accomplished: (i) packet delivery ratio, (ii) end-to-end delay (in seconds), (iii) “throughput” (in bps), (iv) packet loss rate, (v) “detection rate (DR)”, (vi) “false positive rate (FPR)”.

#### 6.3.1. Effect on Packet Delivery Ratio

**Figure 10:**
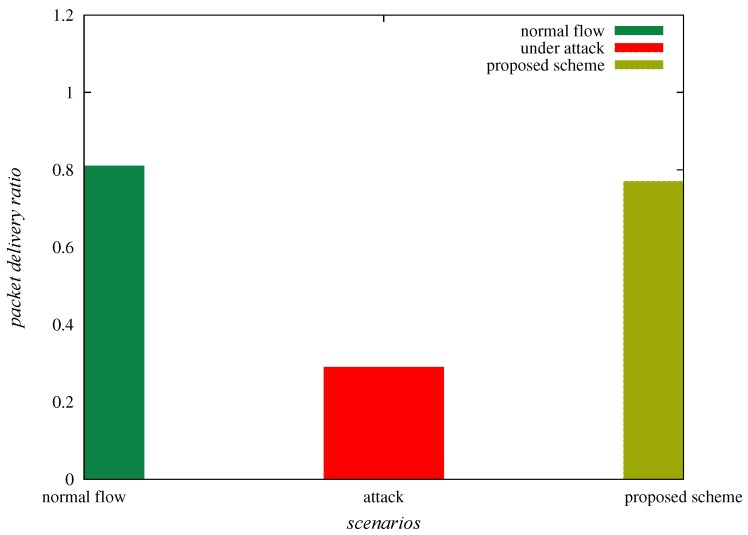
Comparison of packet delivery ratios.

The packet delivery ratio is formulated as “the ratio of packets received at the base station to packets transmitted by source nodes” (for example, from $S_{i}$ to $EN_{j}$). Table 4 provides packet delivery ratio in different instances, such as normal flow of traffic, under sinkhole attack and “under deployed SAD-EIoT”. From Table 4 and Figure 10, it is confirmed that the packet delivery ratio for the instances under normal flow of traffic, under sinkhole attack and “under SAD-EIoT” are 0.81, 0.29 and 0.77, respectively. Thus, it is observed that the packet delivery ratio is indubitably improved under the deployment of “SAD-EIoT” as compared to the case of sinkhole attack.

#### 6.3.2. Effect on Packet Loss Rate

**Figure 11:**
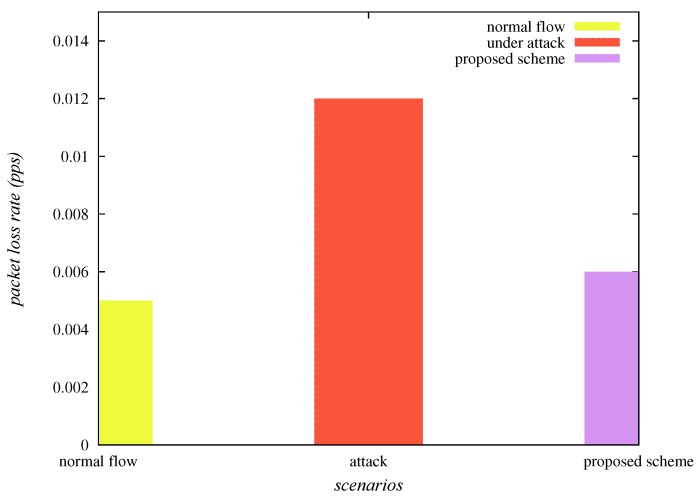
Comparison of packet loss rates.

The packet loss rate is also one of the important network parameters. It is estimated as the “number of packets lost per unit time”. It is required that for a reliable communication of the network, the packet loss rate should be as least as possible. Table 4 and Figure 11 show that the packet loss rate (packets per second pps), under normal flow of traffic, under sinkhole attack and “under SAD-EIoT” are 0.005, 0.012 and 0.006, respectively. Thus, it is observed that the packet loss rate is indubitably reduced under the deployment of “SAD-EIoT” as compared to the case of sinkhole attack.

#### 6.3.3. Effect on End-to-End Delay

**Figure 12:**
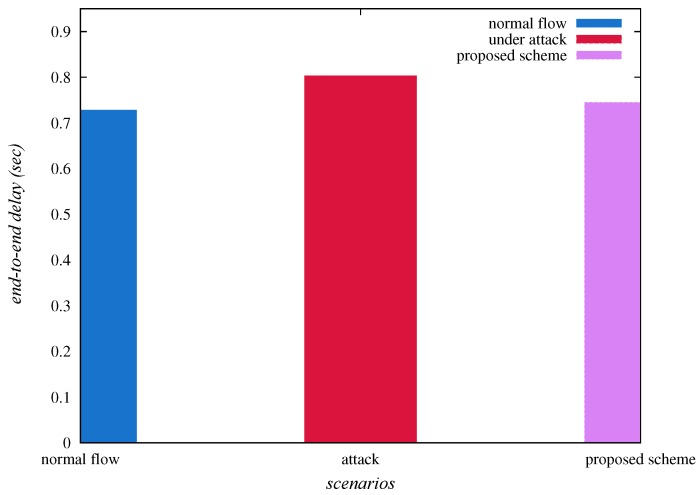
Comparison of end-to-end delays.

The end-to-end delay is estimated as “the average time taken by the data packets to arrive at the base station, for example, $EN_{j}$ from $S_{i}$”. Table 4 represents the “end-to-end delay (in seconds)”, under normal flow of traffic, under sinkhole attack and “under the deployment of SAD-EIoT”. Table 4 and Figure 12 confirm that the end-to-end delay, under normal flow of traffic, under sinkhole attack and “under SAD-EIoT” are 0.72803, 0.80338 and 0.74485, respectively. Thus, it is cleared that the end-to-end delay is indubitably reduced under the deployment of “SAD-EIoT” as compared to the case of sinkhole attack.

#### 6.3.4. Effect on Throughput

**Figure 13:**
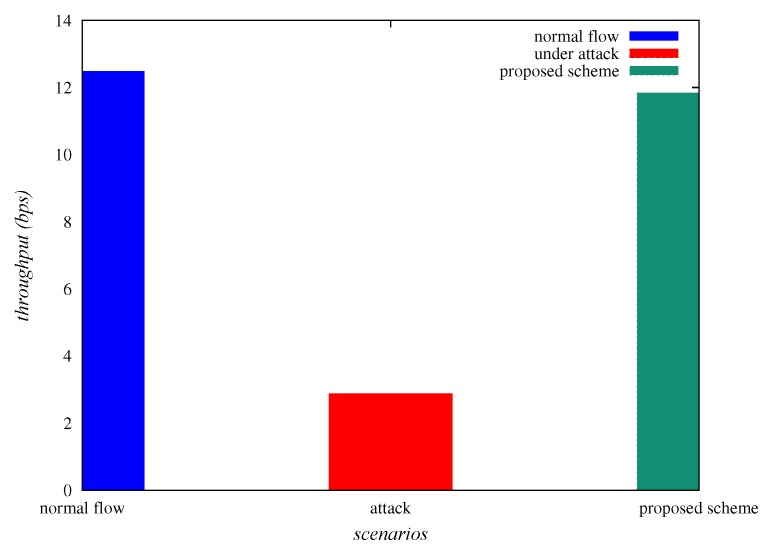
Comparison of throughput.

Throughput is “the number of bits transferred per unit time”. Table 4 represents the throughput (in bps), under normal flow of traffic, under sinkhole attack and “under the deployment of SAD-EIoT”. From Table 4 and Figure 13, it is confirmed that the “throughput”, under normal flow of traffic, under sinkhole attack and “under SAD-EIoT” are 12.48, 2.88 and 11.84, respectively. Thus it is observed that the end-to-end delay is indubitably improved under the deployment of “SAD-EIoT” as compared to the case of sinkhole attack.

The diverse statistics of SAD-EIoT as per the various scenarios are provided in Table 4.

#### 6.3.5. Effect on Detection Rate and False Positive Rate

The other essential performance parameter of an IDS is the DR (which is also known as “true positive rate (TPR) or sensitivity or hit rate” or “false positive rate or fall out (FPR)”). It is very essential to consider this parameter in the evaluation of performance of a proposed intrusion detection scheme. DR can be estimated as “the number of attackers detected by an IDS divided by the total number of attackers present in the test sample” which is formulated as

$$DR=\frac{TP}{TP+FN},$$

whereas FPR is estimated as “the number of nodes falsely detected as attacker nodes” which is formulated as

$$FPR=\frac{FP}{TN+FP}.$$

The obtained results are briefed as follows:

- A confusion matrix of the obtained results is constructed and its details are provided in Table 5. The provided matrix clears that SAD-EIoT detects 23 SHAs.
  Thus, there is a total of 23 TP nodes (actual attackers), one FP node (normal nodes), 96 TN nodes (normal nodes) and one FN node (actually an attacker but identified as a normal node).
- There are in total 24 SHAs and 81 normal nodes. Accordingly, DR and FPR are 95.83% and 1.03% respectively.

## 7. Comparative Analysis of SAD-EIoT with Other Related Existing Schemes

In this part of the paper, the results of SAD-EIoT are compared with other closely related schemes proposed by Salehi et al. [31], Wang et al. [32], Wang et al. [33], Wang et al. [34], Wazid et al. [17] and Wazid et al. [18]. The comparative analysis of outcomes is presented in Table 6. The following observations have been made:

- The DR for Salehi et al.’s scheme [31], Wang et al.’s scheme [32], Wang et al.’s scheme [33], Wang et al.’s scheme [34], Wazid et al.’s scheme [17], Wazid et al.’s scheme [18] and SAD-EIoT are 93.00, 90.96, 86.00, 83.00, 95.00, 95.00 and 95.83, respectively.
- The “false positive rate (FPR)” for Salehi et al.’s scheme [31], Wang et al.’s scheme [32], Wazid et al.’s scheme [17], Wazid et al.’s scheme [18] and SAD-EIoT is 10.00, 2.06, 1.25, 1.23 and 1.03, respectively.

Therefore, the designed SAD-EIoT performs better than other related existing schemes.

The comparison of computational complexities of existing schemes and SAD-EIoT is provided in Table 7. The computational complexities for the schemes of Salehi et al. [31], Wang et al. [32], Wang et al. [33], Wang et al. [34], Wazid et al. [17], Wazid et al. [18] and SAD-EIoT are $O(n^{2})$, $O(n^{2})$, $O(n^{2})$, $O(n^{2})$, $O(n^{2})$, $O(n^{2})$ and O(n) respectively. Here, *n* denotes the total number of deployed IoT sensing nodes or sensor nodes in the specified area. The complexities for other existing schemes are quadratic whereas for SAD-EIoT they are linear. Hence, SAD-EIoT performed better in terms of computational costs.

## 8. Concluding Remarks

As discussed in this work, the performance of edge-based IoT communication degrades very rapidly under the presence of various sinkhole attacker nodes (SHAs). Most of the existing schemes for sinkhole node detection are not effective as they cannot identify all possible types of SHAs in EIoT. Moreover, the existing intrusion detection schemes have other limitations, such as inefficiency in terms of communication and computation costs. To overcome this problem, an efficient intrusion detection scheme for the detection of various kinds of SHAs in EIoT (SAD-EIoT) is proposed. SAD-EIoT requires a lower number of exchanged messages that further causes reduction in overall communication cost. Furthermore, SAD-EIoT achieves around 95.83% detection rate and 1.03% false positive rate, which is considerably better than other related existing schemes. The performed security analysis also confirms the resilience of SAD-EIoT against sinkhole attack. Apart from these characteristics, in SAD-EIoT, the resource-constrained IoT devices (sensors) need less computation and communication costs because the resource-rich edge node only executes the steps of SHAs detection algorithm. Eventually, SAD-EIoT will be a suitable match for those applications which can be used in critical and sensitive operations (for example, surveillance, security and monitoring systems).

In the future, a testbed for the proposed SAD-EIoT scheme along with detection of a greater number of attacks, such as blackhole, greyhole, wormhole and other routing attacks in IoT environment can be implemented and analysed.

## Figures and Tables

**Table 1:** Comparison of existing techniques.

Protocol	Goal	Method Used	Outcomes and Limitations
Wang et al. [34]	Intrusion detection	Single and multi sensing detection methods	Performed intrusion detection with low detection rate
Wang et al. [32]	Intrusion detection	Misuse based IDS method	Performed intrusion detection with low detection rate along with high computational cost
Wang et al. [33]	Intrusion detection	Gaussian and uniformly distributed method	Performed intrusion detection with low detection rate (in the case of lower number of nodes)
Salehi et al. [31]	Intrusion detection	Information flow based detection	Performed intrusion detection with high false positive rate
Wazid et al. [17]	Sinkhole node detection in WSN	Cluster based sinkhole node detection	Performed sinkhole node detection with high computation cost
Wazid et al. [18]	Routing attack detection in IoT	RAD-EI	Performed routing attack detection with high computation cost

**Table 2:** Notations utilised in SAD-EIoT.

Symbol	Description
$EN_{j}$	*j*th edge node
$S_{i}$	*i*th IoT smart device (sensor node)
$SHA_{k}$	*k*th sinkhole attacker node
${⋃}_{l}$	List of suspected SHAs
$SHA_{list}$	List of SHAs
$ID_{S_{i}}$, $ID_{EN_{j}}$	Identities of $S_{i}$ and $EN_{j}$, respectively
$SK_{EN_{j},S_{i}}$	Shared secret key between $EN_{j}$ and $S_{i}$
$REN_{S_{i}}$	Energy remaining value at an IoT sensor $S_{i}$
$R_{S_{i}}$	Rank of an IoT sensor $S_{i}$
$R_{LS_{i}}$, $R_{U_{S_{i}}}$	Lowest and highest values of ranks of $S_{i}$, respectively
$HC_{S_{i}}$	Hop count for $S_{i}$ from $EN_{j}$
$HC_{\theta }$	Threshold value of hop count for the network
$\mu_{sdq}$, $\mu_{sr}$	Messages containing status & data query and status response, respectively
$\mu_{d}$, $\mu_{in}$	Messages containing only data and information, respectively
$sdq_{rq}$, $s_{rp}$	Information contents in messages $\mu_{sdq}$ and $\mu_{sr}$, respectively
data	Message $\mu_{d}$’s data content
WT $WT_{\theta }$	Waiting time and its threshold at $EN_{j}$, respectively
$PD_{n}$, $PD_{a}$, $PD_{s}$	Packet delivery ratios under normal circumstance of traffic,
	attack condition and under SAD-EIoT, respectively
DR	Detection rate/true positive rate (TPR)
FPR	False positive rate
TP, FP	True and false positives, respectively
TN, FN	True and false negatives, respectively
$\nu_{n}$, $\nu_{a}$, $\nu_{s}$	End-to-end delay (in seconds) in normal condition,
	attack condition and under SAD-EIoT cases, respectively
$\Lambda_{n}$, $\Lambda_{a}$, $\Lambda_{s}$	Throughput in bits per second (bps) in normal condition,
	attack condition and under SAD-EIoT cases, respectively
$|\mu_{d}|$	“Total data packets transmitted by IoT sensing devices”
$|\mu_{d^{\prime }}|$	“Total confirmed data packets received at $EN_{j}$”
$|\mu_{dpa}|$	“Total data packets that are not transmitted to edge node” by attacker nodes
$|\mu_{dpa^{\prime }}|$	“Total data packets that are not transmitted to edge node” by authentic attacker nodes (TP)
$|\mu_{d_{1}}|$	“Total data packets that are not transmitted to edge node” by attacker nodes (FN)
$\mu_{in}$	“Information message transmitted by each $EN_{j}$” to all regular IoT sensing nodes
$T_{send_{i}}$, $T_{rec_{i}}$	“Sending and receiving time” of a data packet, say *i*, respectively
h(·)	“One-way collision-resistant cryptographic hash function”
*p*	Total packets
pkt, $pkt_{s}$	A data packet and its corresponding size
*ℏ*	Hashed message authentication code (HMAC)
X||Y	Concatenation of data *X* with data *Y*

**Table 3:** Parameters used in simulations.

Parameter	Description
Platform	Ubuntu 18.04 LTS
Network area	650×250 m${}^{2}$
Number of nodes	121 nodes
Number of attacker nodes	24
Time considered for simulations	1800 s
Traffic type	CBR/UDP
Packet transmission rate	25 Kbps
IoT device’s communication range	100 m

**Table 4:** Obtained statistics of EIoT for different cases.

Parameter	Case of Normal Flow	Case of Sinkhole	Under the Deployment
	of Traffic	Attack	of SAD-EIoT
Packet delivery ratio	0.81	0.29	0.77
Packet loss rate (in pps)	0.005	0.012	0.006
End-to-end delay (in seconds)	0.72803	0.80338	0.74485
Throughput (in bps)	12.48	2.88	11.84

**Table 5:** Confusion matrix for SAD-EIoT.

	Actual Value		
Predicted value		Positives	Negatives
	Positives	TP: 23	FP: 01
	Negatives	FN: 01	TN: 96

**Table 6:** Accuracy comparison among existing schemes and SAD-EIoT.

Scheme	[31]	[32]	[33]	[34]	[17]	[18]	SAD-EIoT
“Detection rate (DR) (in %)”	93.00	90.96	86.00	83.00	95.00	95.00	95.83
“False positive rate (FPR) (in %)”	10.00	2.06	N/A	N/A	1.25	1.23	1.03

*Note:* N/A: not available.

**Table 7:** Comparison of computational complexity among existing schemes and SAD-EIoT.

Scheme	[31]	[32]	[33]	[34]	[17]	[18]	SAD-EIoT
Computational complexity	$O(n^{2})$	$O(n^{2})$	$O(n^{2})$	$O(n^{2})$	$O(n^{2})$	$O(n^{2})$	O(n)

*Note:* N/A: not available.
